# Progress in Surgery

**Published:** 1897-02-27

**Authors:** 


### Progress in Surgery.

### ORTHOPÆDIC SURGERY.

(*Continued from page* 351.)

**Congenital Scoliosis.**—Coville[7] gives the results of his observations and measurements of 1,015 infants. There were only two congenital scoliotic spines in the entire number. One was examined on the eighth day; there was no trace of rickets, and the curvature was convex to the right. The second was examined when sixty-six days old; many signs of rickets were present, and the case could not, therefore be called truly congenital, but was one of fœtal rickets. Cases of congenital scoliosis are very rare indeed. Tubby has recorded four in his recent work on "Deformities." Bouvier and Bouland found this condition only in cases of fœtal rickets, or in monsters. Bush details one case which Coville thinks was a monster. There seem to be three general classes of cases—malformations or derangements; multiple osseous malformations; and, thirdly, the much more obscure type of pure scoliosis.

Crivelli[8] describes a case of lateral deviation of the trunk produced by adhesions of the superior gluteal nerve to an exostosis of the ilium, following an injury resulting from a fall over a bar of iron about five years ago. The patient inclined the whole body to the right, so as to bear his weight upon the right leg and to avoid pressure on the left foot. The pain was so intense that he could scarcely walk at all when he was first seen. On careful examination a painful spot was found at the margin of the great sacro-sciatic foramen just where the superior gluteal nerve passes out of it. This spot was cut down upon, and the superior gluteal nerve was found to be surrounded by a new growth, partly osseous and partly fibrous. The growth was carefully dissected away, and the patient's pain from that time forward was entirely removed.

**Congenital Displacement of the Hip.**—Lorenz[9] writes fully on the bloodless treatment of congenital hip displacement. It would appear that Lorenz has abandoned the open method of operating for this deformity in children under seven. He now follows the manœuvres of Paci, putting the affected limb up in extreme abduction, and maintaining it so for two to four months, and then allowing the patient to walk about in a properly constructed apparatus. Dolega,[10] Mikulicz, and Hoffa also write on the bloodless method of reposition of the head of the femur; and it would appear not to be necessary to perform the operation so frequently as formerly. Gibney[11] records a case of suppuration after the Lorenz operation for this affection. Suppuration set in on the second day, but eventually the parts healed nicely, and the final result showed half an inch of shortening, and no slipping of the head of the bone ten months afterwards. Whitman[12] records also a tolerably successful case of the same affection; it was treated by operation after Lorenz's method. A critical digest of the pathology and general treatment of displacement of the hip is contributed by Mr. Montgomery.[13] He enters very fully into the anatomy of the affection, and discusses the various methods of treatment, and concludes that Paci's manipulations should first be tried, whatever the age of the patient If this do not succeed, Lannelongue's suggestion is safe, viz., the injection of chloride of zinc about the joint. And these failing, Lorenz's operation should be performed. Quénu[14] has had under observation a considerable number of cases, and, like Kirmisson, has come to the conclusion that very many of these cases should not be treated by operative measures.

**Genu Valgum Adolescentium.**—Dr. A. F. Dixon[15] reports a case of adult rickets occurring in a patient at the age of 18 resulting in extreme genu valgum, so that the limbs crossed like the limbs of an X. Section showed that the lower end of the bone as well as the upper part of the tibia were the seat of rhachitic disease, as indicated by the irregularity of the epiphysial cartilage and the porous condition of the diaphysis. The section showed also distinctly that the marked deformity was due chiefly to the condition of the diaphyses of both femur and tibia, and to a slight extent to the unequal growth of the inner and outer parts of the epiphysis.

**Talipes Equino Varus.—The Value of the Rontgen Rays in Diagnosing the Various Forms of Talipes.**—Barwell[16] states that the Röntgen rays confer on surgeons the power of doing with pretty accurate precision what he has hitherto been able to do but tentatively, namely, to plan beforehand the mode and extent of his osseous operation on club foot. A pair of compasses and a goniometer applied *secundum artem* to the diagram will enable the surgeon to fix on the part where some removal of bone will have the best effect, as also on the amount to be excised. His knowledge of surgical anatomy will do the rest.

**The Treatment of Congenital Talipes Equino Varus.**—Kirmisson[17] in cases of medium severity prefers open operations, and, of those he selects that of Phelps. So far he has performed Phelps' operation 76 times on 51 patients, the youngest being 15 months, the oldest 19 years of age. Tenotomy of the tendo Achillis was done in 23 of these patients, one or two of whom had previously been subjected to instrumental tarsoclasis and three to ablation of the astragalus. Professor Berger,[18] in cases of club-foot in older children prefers cuneiform resection. The best results are obtained from Gross's operation, astragalectomy with cuneiform tarsectomy, as the former alone does not sufficiently remedy the twist in the foot.

[7] Revue d'Orthop, 1896, No. 4. [8] Intercolonial Med. Jour. of Australia, Vol I., No. 2. [9] Sammlung Klinischer Vorträge, Nos. 151-52. [10] Centralblatt für Chirurgie, Aug., 1896. [11] Amer. Med. Surg. Bulletin, Mar. 7th, 1896. [12] Ibid. [13] Med. Chronicle, Aug. 1896. [14] Med. Week, May 1st, 1896. [15] Brit. Med. Jour., June 20, 1896. [16] Lancet, July 18, 1896. [17] Med. Week, Oct. 23, 1896. [18] Ibid.

### DISEASES OF THE LARYNX.

**Examination of the Larynx in Young Children.**—Lambert Lack, at the meeting of the London Laryngological Society in February this year, demonstrated a method of examining the larynx which was the more easy of application the younger the child. He passes the left forefinger into the mouth, and with the finger-tip well in the patient's right *vallecula* or pyriform fossa, pulls forward the base of the tongue and with it the epiglottis. The child, of course, is held sitting upright in an attendant's lap, and the head retracted. By this manner one can obtain a complete view of the larynx by means of the laryngoscope used in the usual manner. For children who have cut their teeth he uses a gag, or else substitutes for the finger a flat copper band like a tongue depressor, with the last half-inch abruptly bent down at a right angle with the lingual portion.

M. Escat[1] has likewise described a method of examining the larynx in young children. He employs a laryngeal mirror with a strong shank and a special tongue depressor. His tongue depressor is curved to adapt it to the tongue, and a curved fork with two bulbous-ending prongs which lie *in situ* in the hyoid fossæ is attached to its distal extremity. In using it the child is held in position by two assistants in the same manner as for intubation. The tongue depressor is rapidly passed to the posterior pharyngeal wall, then downwards, and lastly, forwards, so as to pull forward the base of the tongue. The strong laryngeal mirror is immediately afterwards introduced, but for the first few seconds reflex pharyngeal spasm prevents the larynx coming into view; then when the child takes a breath the cords may be seen. The whole operation of the examination should not exceed ten seconds, a second or third trial being usually possible. Needless to say, as the author remarks, the method requires an expert in the use of the laryngoscope. Kirstein has found that the larynx and trachea can be very easily and thoroughly examined in all children deeply under chloroform by means of his autoscope, while it is not difficult to obtain a view of the larynx, or at least its posterior half, in many children without any anæsthetic.

**Innervation of the Larynx.**—At the Berlin Laryngological Society, Grabower[2] reports a case of recurrent paralysis in tabes which he had under observation ten years in conjunction with H. Oppenheim. He considers that this case proves clinically the correctness of his view, discovered experimentally, that the accessorius is not concerned in the motor nerve supply of the larynx, and that the vagus is the sole motor laryngeal nerve. After death they were able to demonstrate in the microscopical preparations from the extra bulbar accessory and vagus roots, that the accessory roots appeared normal, while the vagus roots were much atrophied.

**Eucaine.**—This new local anæsthetic has proved to be a very efficient substitute for cocaine, and having a far less toxic power may be used with greater freedom than cocaine without fear of bad symptoms arising. Jobson, Horne, and Yearsley[3] describe the chemical constitution of this drug, and relate their experiences of its clinical value. Eucaine is the methylester of benzoyl-n-methyl-tetra-methyl-gamma-oxy-piperidine-carboxylic acid. Eucaine base crystallises in large glistening pyramids, which melt at 104C. Eucaine hydrochloride dissolves in about ten parts of water at room temperature. Vinci carried out certain physiological experiments with eucaine on mice, rabbits, and dogs. He found that the instillation into the eye of a rabbit or dog of a 2 to 5 per cent. solution produced complete anæsthesia in one or two minutes, which lasted on an average ten or twenty minutes. A slight hyperæmia was produced. Upon the central nervous system it acts first as an excitant; later on in toxic doses producing paralysis. Small doses increase the reflex excitability of mice and rabbits. Doses of ⅓rd grain per kilo body weight cause tonic and clonic convulsions, which last several seconds, and recur at moderate intervals. An increase of the dose causes paralysis, under which the animal dies. He found that small and moderate doses slow the pulse rate. Berger's investigations corroborated those of Vinci upon all important points.

Horne and Yearsley used solutions of three strengths—2, 5, and 8 per cent. Of these they found that the 2 per cent. solution was quite sufficient for anæsthetising the uvula, &c., for laryngoscopy or posterior rhinoscopy, and for aural examinations. The 5 and 8 per cent. solutions were used for operative procedures. Although in one case an aural furuncle was incised without pain by the 5 per cent., and in four cases this strength was sufficient to permit of myringotomy, it was generally found that for all operative measures on throat, nose, or ear the 8 per cent. solution was more reliable; especially as in their experience eucaine is devoid of all unpleasant after-effects, they consider it of advantage to use the stronger solution. It should be applied in the same way as cocaine is applied. They found that the anæsthesia was slightly slower in onset than that of cocaine, and that the cases required from five to ten minutes to elapse before they were ready for operation. When anæsthesia was established it was fully equal to that of cocaine. The duration of anæsthesia was from ten to twenty minutes. They observed no alteration in pulse rate.

[1] Archives de Laryng, Sept., Oct., 1896. [2] May 8, 1896; Journ. of Laryng., Nov., 1896. [3] B.M.J., Jan. 16, 1897.

